# Epidemiological metrics and benchmarks for a transition in the HIV epidemic

**DOI:** 10.1371/journal.pmed.1002678

**Published:** 2018-10-25

**Authors:** Peter D. Ghys, Brian G. Williams, Mead Over, Timothy B. Hallett, Peter Godfrey-Faussett

**Affiliations:** 1 Joint United Nations Programme on HIV/AIDS, Geneva, Switzerland; 2 South Africa Centre for Epidemiological Modelling and Analysis, Stellenbosch, South Africa; 3 Center for Global Development, Washington, DC, United States of America; 4 Department of Infectious Disease Epidemiology, Imperial College London, London, United Kingdom

## Abstract

Peter Godfrey-Faussett and colleagues present six epidemiological metrics for tracking progress in reducing the public health threat of HIV.

## Introduction

The goal of ‘Ending the AIDS epidemic as a public health threat by 2030’ has been reflected in the Sustainable Development Goals (SDGs), and similar language has been adapted for other diseases and conditions [1]. Between 2010 and 2017, the number of AIDS-related deaths has declined by 34%, and the number of new HIV infections has declined by 18% [2]. Although these declines constitute important achievements, progress has been slower than envisaged, which is likely due to a combination of suboptimal or inappropriate policies, lack of funding, limited or misdirected implementation of available strategies and tools, or other obstacles. Metrics and corresponding target values or benchmarks that demonstrate progress in the AIDS response and its effect on the AIDS epidemic are useful as the world heads towards that goal.

‘Ending the AIDS epidemic’ has not been defined in scientific terms, and it can be seen as a global aspiration in a distant future. Elimination of all new infections does not appear possible in the short and medium term with the tools available today. Metrics that signal medium-term progress and can be applied in countries, subnational entities, and population groups may be particularly valuable, as they can allow for local accountability and target-driven programme management. Achieving a certain benchmark would then herald the gradual reduction in the HIV burden in that community and could help lay the groundwork for a push to end the epidemic. The benchmarks for the metrics discussed in this paper should not be seen as indicative of tipping points, as those are unlikely to exist in the real world for an infection with a long incubation period and with survival being extended by antiretroviral treatment. Rather, they can be seen as important achievements in the management of epidemics.

Metrics for tracking progress towards the end of the epidemic as a public health threat should relate to both technical and popular interpretations of the concept of a ‘public health threat’. HIV threatens affected populations in several ways. First, and most importantly, HIV causes sickness and death, which together contribute to a country’s burden of disease. Second, people living with HIV (PLHIV) and key populations at high risk of infection may be stigmatised, which may constitute an obstacle to effective implementation and warrants ongoing monitoring. Third, the epidemic reduces the size and the productivity of a country’s work force, imposing an indirect economic burden on the affected country. Finally, managing HIV demands health spending by patients, governments, private insurers, and funders, and, in the worst-affected lower-income countries, this fiscal burden can exceed all other health spending, with part of the cost borne by donor countries. Ending the epidemic as a public health threat depends on reducing mortality and incidence by expanding treatment and prevention services.

Here we discuss various HIV epidemic transition metrics that can be used to measure progress in reducing the public health threat of HIV, based on discussions at a meeting convened by UNAIDS in October 2017 in Glion, Switzerland. These metrics have since been included in UNAIDS’ 2018 reports [2,3] for countries and regions. Metrics and benchmarks to track progress in reducing stigma and discrimination—a goal to be pursued on its own merit that also represents a reduction in key determinants of HIV-related service delivery—are also needed and are discussed in a separate paper in preparation for this collection. Relevant and available data for metrics to track progress in reducing stigma and discrimination have also been included in UNAIDS’ 2018 reports [2,3].

## HIV epidemic transition metrics

**Fig 1:**
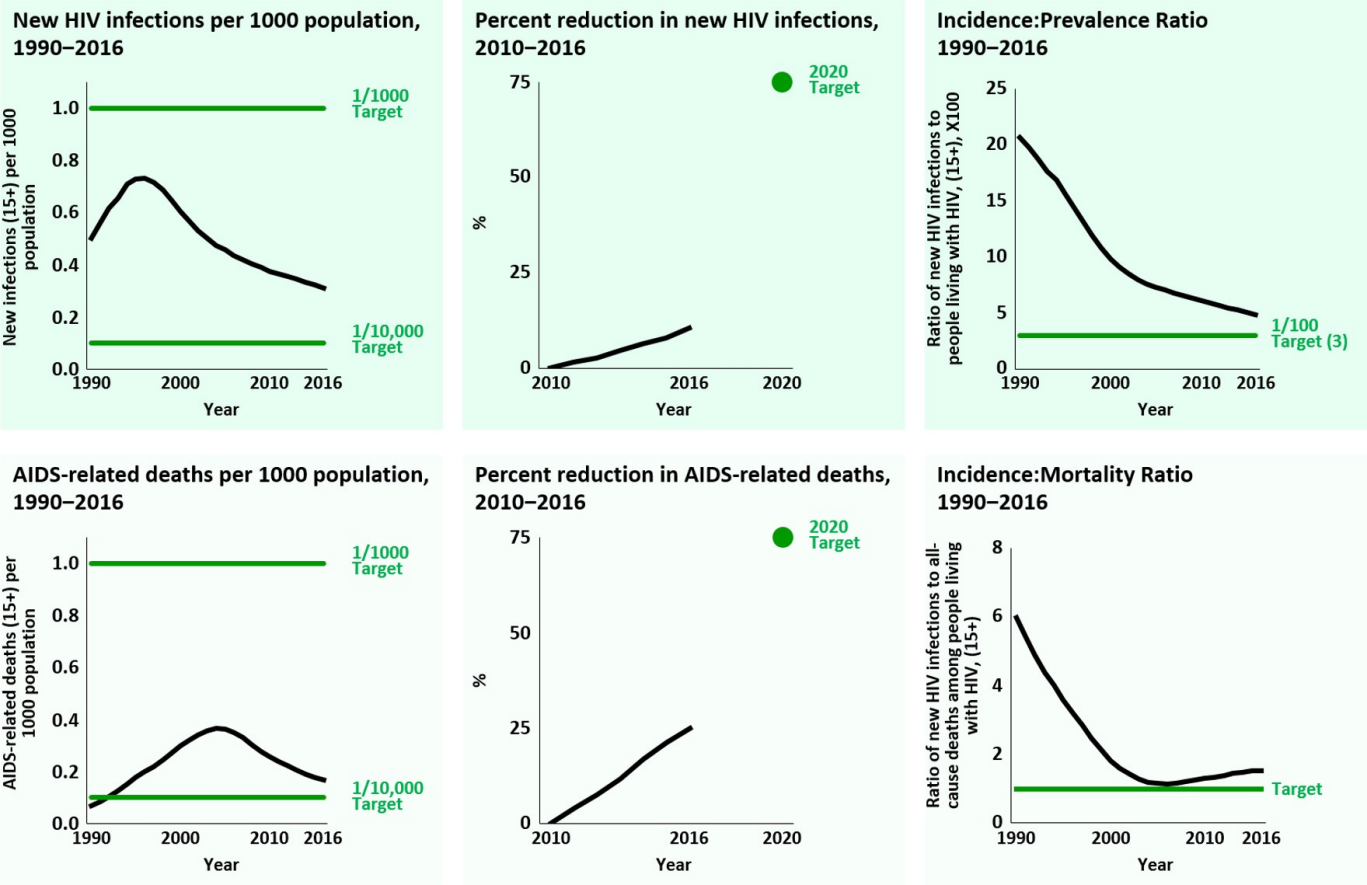
Metrics for HIV transition, globally 1990–2020.

We consider six metrics: (1) an absolute rate of HIV incidence, (2) an absolute rate of AIDS-related deaths, (3) a percentage reduction in new HIV infections, (4) a percentage reduction in AIDS-related deaths [both 3 and 4 as compared to an agreed baseline date], (5) an incidence:prevalence ratio (IPR), and (6) an incidence:mortality ratio (IMR). Fig 1 shows the changing values of the six metrics globally for the percentage reduction during 2010–2016 and for the other metrics during 1990–2016, based on UNAIDS estimates. Key characteristics of the metrics and their benchmark values are shown in Table 1.

**Table 1:** Key characteristics of proposed HIV epidemic transition metrics and their benchmark values.

	HIV incidence rate	AIDS-related mortality rate	HIV incidence reduction compared to 2010: 75% by 2020 and 90% by 2030	AIDS-related mortality reduction compared to 2010: 75% by 2020 and 90% by 2030	Ratio of new HIV infections over PLHIV (IPR)	Ratio of new HIV infections over all-cause mortality among PLHIV (IMR)
Benchmark value	To be set locally	To be set locally	75% by 2020 90% by 2030	75% by 2020 90% by 2030	0.03	1
Complexity	Simple: a single rate in a given year, although the target will need to be setting specific.	Simple: a single rate in a given year, although the target will need to be setting specific.	Intermediate: comparison of two numbers	Intermediate: comparison of two numbers	Complex: ratio between two quantities	Complex: ratio between two quantities (and requires an ancillary condition regarding low mortality)
Use	Adopted as main indicator for monitoring SDG 3.3.1. Regularly used by countries and organisations.	Regularly used by countries and organisations.	Many countries have adopted national targets for 2020.	Few countries have adopted national targets.	Few countries have used [4–9].	PEPFAR has proposed in its strategies [22,23].
Relevance for countries	Metric applies to all countries, but benchmark value changes relative to the level of countries’ incidence rate.	Metric applies to all countries, but benchmark value changes relative to the level of countries’ AIDS-related mortality rate.	Applies to all countries, regardless of number of new infections in 2010.	Applies to all countries, regardless of number of AIDS-related deaths in 2010.	Applies to epidemics of all sizes, regardless of the level of incidence or prevalence.	Applies to epidemics of all sizes, regardless of the level of incidence or mortality.
Rooted in epidemiological theory about the sustainability of transmission?	No—but the threshold can be setting specific and chosen according to empirical evidence on HIV incidence rates.	No—but the threshold can be setting specific and chosen according to empirical evidence on AIDS death rates.	Partially. The targets are derived using a model that represents the potential impact on the epidemic of a suite of interventions.	Partially. The targets are derived using a model that represents the potential impact on the epidemic of a suite of interventions.	Yes	Yes
Impact of target achievement on burdens: a. Sickness, death b. Fiscal burden	a. Eventually reduces b. Eventually reduces	a. Immediately reduces b. Eventually reduces	a. Eventually reduces b. Eventually reduces	a. Immediately reduces b. Eventually reduces	a. Eventually reduces b. Reduces when incidence is lower than all-cause mortality among HIV+ people	a. Immediately reduces* b. Immediately reduces*
Other	No universal benchmark values exist, as they will change from country to country and by population subgroups.	No universal benchmark values exist, as they will change from country to country and by population subgroups.	A 90% reduction in incidence in one place by 2030 could leave incidence at a higher incidence than somewhere else in 2010—i.e., the metric is permissive to global heterogeneity in epidemic levels.	A 90% reduction in AIDS-related mortality in one place by 2030 could leave AIDS-related mortality at a higher level than somewhere else in 2010—i.e., the metric is permissive to global heterogeneity in epidemic levels.	Applies to national or regional populations, not to subgroups of the population for which HIV transmission occurs between the groups, notably sex- and age-defined groups, and to varying degrees key population groups, including sex workers, transgender people, men who have sex with men, and people who inject drugs.	Applies to national or regional populations, not to subgroups of the population for which HIV transmission occurs between the groups, notably sex- and age-defined groups, and to varying degrees key population groups, including sex workers, transgender people, men who have sex with men, and people who inject drugs.

* When associated with an important reduction in mortality as discussed in the text.

Abbreviations: IMR, incidence:mortality ratio; IPR, incidence:prevalence ratio; PEPFAR, President’s Emergency Plan for AIDS Relief; PLHIV, people living with HIV; SDG, Sustainable Development Goal.

### An absolute rate of HIV incidence

The SDG target for HIV (indicator 3.3.1) is monitored by the number of new HIV infections per 1,000 uninfected population, disaggregated by sex, age, and key populations [10]. A numerical target signifying the ‘End of the AIDS epidemic’ for the HIV incidence rate has been proposed at 1/1,000 person-years (py) in South Africa in the original analysis exploring the powerful effects of antiretroviral treatment in reducing incidence [11]. More recently, the same target value of 1/1,000 py has also been suggested for other high-burden countries [12,13].

However, incidence rates are very different in different populations. A target incidence level for a country with high historical incidence, such as the countries in southern Africa, may not be relevant for a country with historically lower incidence. For example, the incidence in India, taken as a whole, was estimated to be 0.10 (0.08−0.14) / 1,000 py in 2016 [14], and it has never exceeded the 1/1,000 py proposed benchmark [15]. Hence, meaningful targets can only be set in countries and local jurisdictions.

While an incidence rate has been proposed for monitoring the SDG target, both the incidence rate and the number of new HIV infections can be monitored. It is important to realise that, in circumstances of important population growth, absolute numbers of new HIV infections could be increasing while the incidence rate is declining.

### An absolute rate of AIDS-related deaths

The level of AIDS-related mortality varies among countries, within countries, by age, and by population groups. AIDS-related mortality in South Africa, for example, peaked in 2006 when 42% of all deaths were attributable to HIV and the AIDS-related mortality rate was approximately 5/1,000 population [14,16,17]. While an AIDS-related mortality of less than 1/1,000 per year may be relevant for the high-prevalence countries in southern Africa, meaningful targets can only be set in countries and local jurisdictions. The proposed indicator for monitoring AIDS-related mortality is the number of AIDS-related deaths per 1,000 population. As above for the incidence metric, both the AIDS-related mortality rate and the number of AIDS-related deaths can be monitored. In the case of important population growth and a decline in mortality, the rate would decline faster than the number of AIDS-related deaths.

### A percentage reduction in the number of new HIV infections

For the purpose of the Fast-Track modelling, which aimed to develop programmatic targets and resource needs estimates [18], ‘Ending the AIDS epidemic as a public health threat’ was interpreted as achieving an important reduction in incidence and mortality. Programmatic targets and effect estimates were included in the model for testing, treatment, and viral suppression; condom use; key populations (men who have sex with men, sex workers, people who inject drugs, transgender people, and prisoners) intervention packages; preexposure prophylaxis; voluntary male medical circumcision; and conditional cash transfers. Specifically, the percentage reduction target was set to a 90% reduction in the number of new HIV infections in the entire population between 2010 and 2030. The modelling of the impact resulting from reaching the different programmatic targets achieved a reduction by 2030 of nearly 90% [18]. The 2016 UN high-level meeting adopted values of 90% reduction by 2030 and the interim target of 75% for the year 2020, as well as the corresponding programmatic target values [19].

### A percentage reduction in the number of AIDS-related deaths

As for the above percentage reduction in new HIV infections, a 90% reduction in AIDS-related deaths was aimed for in the Fast-Track modelling between 2010 and 2030 [18]. The modelling of the impact resulting from reaching the different programmatic targets achieved a nearly 80% reduction by 2030 [18]. The 2016 UN high-level meeting adopted a target of 90% reduction by 2030 and 75% by 2020 for AIDS-related mortality, together with the corresponding programmatic target values [19].

### An IPR

The IPR (incidence:prevalence ratio) is defined as the number of new infections occurring per year in a population divided by the number of persons living with HIV in that same population. When the IPR is below a certain threshold, the epidemic declines—if the IPR is held below that threshold for a long time, the epidemic will eventually be eliminated. The IPR is thus an indicator for the epidemic response ‘proceeding in the right way’, as opposed to it having achieved its goal of ending the AIDS epidemic. It was initially used by researchers in high-income countries [20,4,5], relating it to the effective epidemic number and using it to judge progress in the epidemic and the response. More recently, it has been used to judge progress in the response to the United States epidemic [5–7].

A global benchmark value is obtained by considering the replacement level for the infection: for an epidemic to decline, on average, there should be fewer than one new infection per person living with HIV over the course of their infection. If we assume that newly infected persons survive for D years and there is an equal risk of transmission over this time and that it is the same for all people, then it follows that the benchmark value is equal to 1/D at equilibrium. Making further simplifying assumptions, we can say that D is approximately 33 years (the approximate survival time, accounting for the proportion of PLHIV that benefit from antiretroviral therapy [ART] under UNAIDS’ treatment targets) [9], and so the threshold may be approximately 0.03. We note that this provides only a rough ‘rule of thumb’; a separate paper is in preparation for this collection to explore further the behaviour of the IPR. As the IPR contains elements related to incidence reduction as well as to extending survival, it conveys two major aims of the programmatic response.

### An IMR

The IMR (incidence:mortality ratio) is defined as the ratio of the number of people who become HIV infected per year to the number of people among those already infected who die (from any cause) per year. When this number is greater than one, the size of the population of PLHIV grows; when it is less than one, the size of that population shrinks [21,22].

A target of IMR < 1 on its own does not require a reduction of mortality. In fact, dropping below IMR < 1 is possible with a high level of mortality. An additional target therefore needs to be considered in conjunction with the IMR target [21]. A possible target for a direct mortality reduction is that mortality among PLHIV should approach mortality among the HIV-negative population (which will be country and population specific). Indirect targets could relate to ART coverage—for example, ART coverage > 70% [23] or ART coverage > 81% by 2020 and >86% by 2030 [18,24].

There is a close relationship between the IPR (discussed above under the IPR section) and the IMR. At equilibrium, the two metrics become equivalent if the IMR < 1 criterion provides for the mortality rate being below the same level as that implied by the IPR threshold (1/D).

## Measurement issues

Questions regarding measurement arise for each of the proposed metrics, as the availability of empirical data is a challenge for all proposed metrics.

### Modelled estimates versus empirical estimates

There is a long history of countries developing modelled estimates of HIV incidence, HIV prevalence, AIDS-related mortality, and all-cause mortality among PLHIV. A major process is undertaken by UNAIDS and partners that involves soliciting expert advice about new model features, software development, capacity building in modelling, and annual country-level development of modelled estimates [25]. This results in the most widely available and consistent set of country-level estimates and time trends in the incidence and prevalence of HIV and HIV-related mortality. In addition, several academic groups are active in this field and publish topic- or country-specific models and estimates [16,26,27] or global and country estimates [28].

Clearly, the quality of modelled estimates depends on the availability and validity of input data. Mathematical models that estimate HIV incidence and AIDS-related mortality may include, besides demographic data and assumptions, the following empirical data: sentinel surveillance prevalence among pregnant women, programmatic data on prevalence among pregnant women, national survey–derived HIV prevalence, numbers of people on ART, national survey–derived incidence, cohort-derived incidence among 15–49 year olds, case reports of new HIV diagnoses, case reports of AIDS-related deaths, and coverage and regimen distribution for prevention of mother-to-child transmission (PMTCT). Data-informed assumptions are typically made about the survival probability of people receiving ART, the effectiveness of antiretroviral regimens to prevent MTCT, and other parameters [29]. Modelled estimates are typically produced at the national level and increasingly for subnational geographic entities: however, currently they typically are not available for specific key population groups.

The major challenge with model-based estimates is that they tend to be most uncertain (least precise) in recent years and are, by necessity, dependent on assumptions for which direct empirical data are lacking. When comparing, for example, incidence and mortality, two quantities are being compared, both of which are being modelled from a dataset that is predominantly informed by trends in HIV prevalence and ART coverage. This can be addressed by using the models to ‘test’ whether there is evidence of a particular target being reached or not, in which the uncertainty attendant in the methods is fully expressed. It may be the case that with current surveillance systems and data availability, one may not be able to make very precise statements regarding meeting particular thresholds as soon as we would like. However, new development in the availability of empirical data—especially measures of incidence and deaths—will bolster this ability.

### Empirical data on HIV incidence, AIDS-related mortality, all-cause mortality among PLHIV, and HIV prevalence

Empirical data on HIV incidence are increasingly available but remain rare. Cohort studies that measure the incidence rate directly are costly and time-consuming and may suffer from participation bias [30]. Hence, only a few national cohort studies exist [31,32], with other studies focused on entire populations or subpopulations in specific locations [33,34]. Recently, the cross-sectional population-based HIV impact assessment surveys [35] have been used to generate estimates of HIV incidence (using algorithms that include assays to estimate the number of recent infections) at the national level, although not at the subnational level or among specific key populations. As are cohort studies, these surveys are also subject to participation bias. Separate cross-sectional surveys among key populations are now also recommended to include the measurement of incidence [36], although the body of empirical measurements is currently limited. For both national surveys and surveys among key populations, having a sample size that is large enough to estimate HIV incidence with precision is costly, and this will become even more so as the level of incidence falls to lower values in future years. Nevertheless, the assay-based methods for the measurement of incidence in cross-sectional surveys continue to be investigated and developed [37], and when this information is combined (in models) with other data, the precision of model-based incidence estimates can be improved. Countries with well-developed case-reporting systems can use the annual number of new diagnoses to estimate HIV incidence taking into account other information on the time to diagnosis, underdiagnosis, and failure to report [38,39]. Efforts are ongoing in other countries (including those with the highest levels of prevalence) to develop case-based surveillance and patient-monitoring systems [40]—it is currently unclear whether such systems will allow for the estimation of incidence among key populations.

Comprehensive empirical data on mortality among PLHIV are rare, especially in high-prevalence countries. In countries with well-developed case-reporting systems, data are recorded on all deaths among PLHIV who are diagnosed. In these systems, a proportion of these deaths are registered among people who were not previously known to be living with HIV. Data on all-cause mortality among key populations living with HIV are not available for most countries, although countries with well-developed case-reporting systems that include reporting of the assumed mode of transmission may be able to make estimates based on reported data [39]. In countries with high prevalence of HIV and high mortality among PLHIV, the overall level and the age- and sex-specific patterns of all-cause mortality may be used to constrain modelled estimates [41]. Comprehensive empirical data on AIDS-related mortality specifically are even rarer, especially in countries with a generalised epidemic. Failure to correctly record AIDS-related deaths may be due to stigma [17,42]. In addition, registers may contain the number of PLHIV who have died, but the vital status of individuals lost to follow-up may not be up to date. In countries with well-developed vital registration systems, the failure to correctly record AIDS-related deaths may be due to misdiagnosis of the cause of death [43].

Empirical data on HIV prevalence are widely available. HIV prevalence data are generated for key populations (typically only in specific locations) and for pregnant women in special surveys or through routine reporting systems, especially in countries with a generalised epidemic, and for the entire population from national surveys such as the Demographic and Health surveys [44] and, more recently, the population-based HIV impact assessments [35].

## Conclusion

Much progress has been achieved in recent years in reducing HIV incidence and AIDS-related deaths. In addition to the previously widely used metrics of incidence and mortality rates, additional metrics that show percentage reductions or that relate the number of new infections to either the total number of PLHIV or their all-cause mortality are useful to measure transitions in national or local epidemics. These metrics are now increasingly being used, including by UNAIDS and by the President’s Emergency Plan for AIDS Relief (PEPFAR) [2,3,23]. The different metrics convey information about different aspects of the epidemic: metrics that focus on incidence reductions centre on curbing epidemic spread such that reductions in sickness, death, and fiscal burden will follow later; metrics that focus on reducing the death rate speak to whether there is an immediate reduction in sickness and death; and the metrics that relate to the size of the epidemic speak to whether the fiscal burden is immediately contracting (IMR) or whether the response is ‘on track’ to reduce the fiscal burden (IPR)

The aim of the AIDS response in each local epidemic will be to reach the metric’s benchmark corresponding to epidemic transition and maintain the metric as low as possible below this benchmark level. As the epidemic recedes, the response will need to adapt to the epidemic becoming concentrated in specific population groups, taking into account their specific social and biological characteristics.

Progress in achieving HIV transitions is increasingly going to depend on effectively reducing stigma and discrimination, as they are a key determinant of effective service delivery. Hence, specific tracking of progress in this area is required [2,3].

Given the importance of targets for accountability and programme management, and given the dearth of empirical data, especially on HIV incidence and on mortality, more investment in generating, compiling, reporting, and analysing empirical data is essential.

## Abbreviations

ART: antiretroviral therapy
IMR: incidence:mortality ratio
IPR: incidence:prevalence ratio
PEPFAR: President’s Emergency Plan for AIDS Relief
PLHIV: people living with HIV
PMTCT: prevention of mother-to-child transmission
py: person-years
SDG: Sustainable Development Goal
